# Comparative lipidomic profiling of the human commensal bacterium *Propionibacterium acnes* and its extracellular vesicles†

**DOI:** 10.1039/c7ra13769a

**Published:** 2018-04-23

**Authors:** Jinseong Jeon, Seung Cheol Park, Jin Her, Jae Won Lee, Jin-Kwan Han, Yoon-Keun Kim, Kwang Pyo Kim, Changill Ban

**Affiliations:** Department of Life Sciences, Pohang University of Science and Technology (POSTECH) 77, Cheongam-Ro, Nam-Gu Pohang Gyeongbuk 37673 Republic of Korea; Department of Applied Chemistry, College of Applied Science, Kyung Hee University Yongin 17104 Republic of Korea; Department of Chemistry, Pohang University of Science and Technology (POSTECH) 77, Cheongam-Ro, Nam-Gu Pohang Gyeongbuk 37673 Republic of Korea ciban@postech.ac.kr; Myeongdong Medical Woori Technology Building, World Cup buk-ro 56-gil 9, Mapo-gu Seoul 03923 Republic of Korea

## Abstract

*Propionibacterium acnes* is a lipophilic commensal bacterium mainly found on the skin and in the gastrointestinal tract. Pathophysiological effects of *P. acnes* have recently been reported not only in acne progression but in various diseases. As an emerging mode of bacterial communication, extracellular vesicles (EVs) have been demonstrated to conduct critical pathophysiological functions. To provide information on *P. acnes* lipid composition for the first time, we conducted a comparative lipidomic analysis of *P. acnes* and *P. acnes* EVs and identified 214 lipids with high confidence using triplicated liquid chromatography coupled to tandem mass spectrometry (LC-MS/MS) analyses. *P. acnes* EVs contained substantially more PCs, DGs, PAs, PEs, LPAs, LPCs, and MGs than *P. acnes*, and contained fewer PSs, SO1Ps, SA1Ps, LPGs, LPIs, and LPSs. Distinctively, *P. acnes* EVs possessed a markedly reduced amount of TG. These findings will provide useful clues for understanding the biological and pathophysiological mechanisms of *P. acnes* and for clinical applications such as vaccine development, diagnostics and therapeutics.

## Abstract 2

*Propionibacterium acnes* is a lipophilic commensal bacterium mainly found on the skin and in the gastrointestinal tract. Pathophysiological effects of *P. acnes* have recently been reported not only in acne progression but in various diseases.

## Graphical abstract

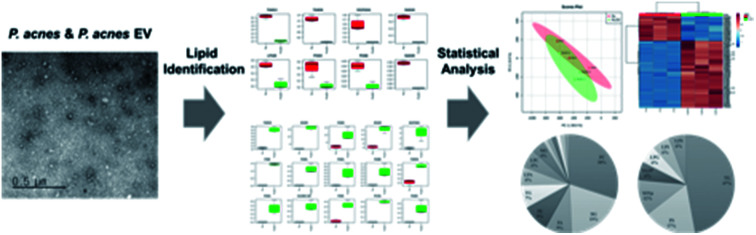

## Introduction

*Propionibacterium acnes* is a lipophilic commensal bacterium found on the skin and in the gastrointestinal tract, and has recently been demonstrated to be an underestimated pathogen in various diseases.^1,2^*P. acnes* is known to be beneficial in normal quantities,^3^ although it is also well characterized as a direct instigator of inflammation in acne, as its secreted products are a significant factor in the etiology of acne.^4,5^ Several studies have recently reported that *P. acnes* can cause not only acne but various other diseases, such as chronic prostatitis leading to prostate cancer; synovitis, acne, pustulosis, hyperostosis, and osteitis (SAPHO) syndrome; sarcoidosis; sciatica; and implant-associated infections.^2^ To understand the etiological role of *P. acnes* in these diseases, it is important to analyze its secretions, which lead to host inflammatory responses and lipogenesis.

Extracellular vesicles (EVs) are an emerging mode of intercellular communication found in all domains of life.^6^ EVs are lipid bilayer-enveloped organelles of 20–1000 nm diameter that contains various biomolecules such as DNA, RNA, proteins, and lipids.^7,8^ EVs are thought to be a major transportation system for extracellular lipids.^9^ Because of their nanoscale size, it is thought that EVs may transfer their cargo in high concentrations to microenvironments that the bacterium of origin cannot reach.^10^ Moreover, EVs have been shown to play a role in pathogenesis, so research on pathogenic bacteria and their EVs should ideally be performed concurrently.^11^ Therefore, the isolation of *P. acnes* EVs and the identification of their lipid components may provide novel insights into the pathophysiological role of *P. acnes* and potential targets for the development of diagnostics and vaccines against *P. acnes*.

Liquid chromatography coupled to tandem mass spectrometry (LC-MS/MS) is a powerful tool for analyzing lipid classes in biological samples.^12–14^ Moreover, the introduction of ultra-performance liquid chromatography (UPLC) allows the rapid and effective separation of individual lipid species.^15^ Multiple reaction monitoring (MRM) based on triple quadrupole (QqQ)/MS can also be used for sensitive and precise quantitative analysis of target compounds.^16,17^ Optimized UPLC-QqQ/MS-based lipidomics analysis has been used to provide accurate information on a wide variety of biological samples and broaden our understanding of many biological events.^18–20^

In this study, UPLC-QqQ/MS was used to profile lipids from *P. acnes* and its EVs for the first time. The goal of this paper was to suggest that *P. acnes* EV was an active biomolecule and to report the comparative lipid composition of *P. acnes* and *P. acnes* EV for the first time.

## Experimental

### Bacterial cell culture and EV isolation

*P. acnes* cultivation and EV isolation were performed as previously described.^21^ Briefly, the *P. acnes* 6919 type strain was cultured in brain heart infusion (BHI) broth at 37 °C in an anaerobic chamber (90% N_2_ and 10% CO_2_). The culture media were harvested at mid-exponential phase (OD_600_ = 1.0–1.5), centrifuged at 10 000 × *g* for 15 min at 4 °C, and then filtered through a 0.45 μm bottle-top filter. After ultrafiltration with a QuixStand benchtop system (GE Healthcare, UK) using a 100 kDa hollow fiber membrane, the concentrate was filtered through a 0.22 μm bottle-top filter. *P. acnes* EVs were purified from the final filtrate by ultra-centrifugation at 150 000 × *g* for 3 h at 4 °C and density gradient ultracentrifugation with 10%, 40%, and 50% OptiPrep solutions (Axis-Shield PoC AS, Norway) at 200 000 × *g* for 2 h at 4 °C. The diluted OptiPrep solutions were prepared in HEPES-buffered saline (20 mM HEPES, 150 mM NaCl, pH 7.4). The purified *P. acnes* EVs were reconstituted in Dulbecco's phosphate-buffered saline (DPBS), quantified by bicinchoninic acid (BCA) assay, and divided into several aliquots, which were stored at −80 °C until use.

### Transmission electron microscopy (TEM) and dynamic light scattering (DLS) analysis

Purified *P. acnes* EVs were characterized using TEM and DLS analysis. For TEM analysis, a single droplet of 100 μg mL^−1^*P. acnes* EVs was loaded onto a 400-mesh copper grid. The grid was allowed to absorb the *P. acnes* EVs for 5 min, then washed with deionized water and negatively stained using 2% uranyl acetate. TEM images were obtained using a JEM-1011 transmission electron microscope (JEOL, Japan) at an accelerating voltage of 100 kV. The average size of *P. acnes* EVs was determined using a Zetasizer Nano series instrument (Malvern Instruments, UK) equipped with a 633 nm laser line at a scatter intensity of 10 × 30 s.

### Mammalian cell culture

The murine macrophage cell line Raw264.7 was obtained from Korean Cell Line Bank (Seoul, South Korea). Cells were cultured in Dulbecco's modified Eagle's medium (DMEM) supplemented with 10% heat-inactivated fetal bovine serum (FBS), 100 U mL^−1^ penicillin, and 100 μg mL^−1^ streptomycin. Cells were incubated at 37 °C in a 5% CO_2_ humidified incubator.

### Analysis of *in vitro* cytokine production

The expression of pro-inflammatory cytokines from mouse peritoneal macrophages (RAW264.7 cells) was measured by enzyme-linked immunosorbent assay (ELISA; R&D Systems, USA) in accordance with the manufacturer's instructions. First, 2 × 10^5^ cells were seeded in 12-well plates. After 24 h, the cells were incubated with 10–1000 ng mL^−1^*P. acnes* EVs for 6–24 h. After incubation, the culture media supernatants were collected, and residual cells and debris were removed by centrifugation at 5000 × *g* for 5 min.

### Lipid extraction

All lipid standards were dissolved in methanol, stored at −27 °C, and diluted to the required concentrations for spiking. A modified two-step extraction method, designed for the high efficiency extraction of non-polar and polar lipids, was performed.^22^ First, for non-polar lipid extraction, 990 μL of methanol/chloroform (2 : 1, v/v) and 10 μL of 1 μg mL^−1^ lipid standards including TG (11 : 1–11 : 1–11 : 1), DG (8 : 0–8 : 0), MG (15 : 1), ChE (10 : 0), PC (10 : 0–10 : 0), LPC (13 : 0), PE (10 : 0–10 : 0), LPE (14 : 0), PS (10 : 0–10 : 0), LPS (17 : 1), PG (10 : 0–10 : 0), LPG (14 : 0), PI (8 : 0–8 : 0), LPI (13 : 0), PA (10 : 0–10 : 0), LPA (14 : 0), So (d17 : 1), So1P (d17 : 1), Sa (d17 : 0), Sa1P (d17 : 0), SM (d18 : 1–12 : 0), Cer (d18 : 1–12 : 0), dCer (d18 : 1–12 : 0), Cer1P (d18 : 1–12 : 0), and dCer1P (d18 : 1–16 : 0) were added to the cell pellet. The sample was vortexed intensely for 30 s every 3 min and centrifuged (14 000 × *g*, 2 min, 4 °C). The supernatant was moved to a new tube. Second, for polar lipid extraction, 750 μL of methanol/chloroform/37% (1 N) HCl (80 : 40 : 1, v/v/v) was added to the remaining pellet and incubated for 15 min at room temperature with vortexing every 5 min for 30 s. After 15 min, the tube was moved to ice and an additional 250 μL of cold chloroform and 450 μL of 0.1 M cold HCl were added. After vortexing for 1 min, the tube was centrifuged (6500 × *g*, 2 min, 4 °C) and the lower organic phase was transferred to a new tube.

### Trimethylsilyldiazomethane (TMSD) methylation

For TMSD methylation, lipid extracts were dried and dissolved in 200 μL methanol. TMSD (2 M, dissolved in hexane) was added to the lipid extracts and the samples were vortexed for 30 s and incubated for 15 min at 37 °C. The reaction was quenched with acetic acid (10 μL) and the yellow solution became colorless. The samples were injected into the LC/MS.^16^

### LC-MS analysis

For the quantitative profiling of lipids, sequential UPLC-QqQ MS analysis was constructed using MRM. The UPLC (1290 infinity, Agilent Technologies, USA) system was composed of a binary pump (G4220A, USA), an autosampler (G4226A, USA), and a column compartment (G1316C, USA). Samples were injected into the sample loop and separated by a Hypersil GOLD column (2.1 × 100 mm, 1.9 μm, Thermo Science) with a linear gradient of solvents A (19 : 19 : 2 acetonitrile : methanol : water, 20 mM ammonium formate, 0.1% formic acid) and B (2-propanol, 20 mM ammonium formate, 0.1% formic acid). The temperatures of the sampler and column oven were set to 4 and 40 °C, respectively. A 33 min gradient was performed as follows: 0–5 min with 5% B, 5–15 min with 5–30% B, 15–22 min with 30–90% B, 22–27 min with 90% B, 27–28 min with 90–5% B, and 28–33 min with 5% B. The flow rate, 250 μL min^−1^, was adjusted for all gradient times and the injection volume for each run was 4 μL. For MS analysis, an Agilent 6490 Triple Quadrupole mass spectrometer (Agilent Technologies, USA) was used, with the following parameters: 4000 V positive mode capillary voltage, a sheath gas flow of 11 L min^−1^ (ultra high purity nitrogen), a drying gas flow of 15 L min^−1^ at various temperatures, and a nebulizer gas flow at 25 psi. Optimized MRM conditions were used to analyze the various lipid species.

### Data processing and statistical analysis

LC-MS data were obtained and processed using Agilent Mass Hunter Workstation Data Acquisition software. MRM data on the target lipids, including the *m*/*z* of precursor and product ions, and the retention time were exported using Qualitative Analysis B.06.00 software (Agilent Technologies, USA). Next, an in-house database constructed using the Skyline software package (MacCoss Laboratory, USA) was applied to determine the peak area of assigned lipids from replicate raw data. The extracted areas of lipid species were normalized to the appropriate internal standard. Principal component analysis (PCA) was performed using the MetaboAnalyst website.^23^

## Results

### *P. acnes* EV isolation

**Fig. 1:**
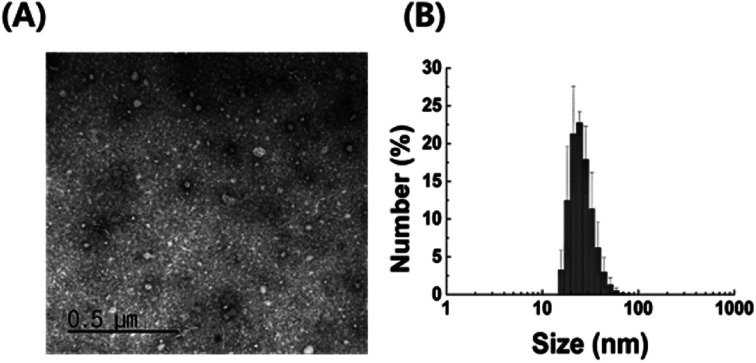
Characterization of EVs isolated from *P. acnes* culture medium (A) TEM image and (B) DLS analysis of purified *P. acnes* EVs.

We examined the characteristics of *P. acnes* EVs purified by serial ultracentrifugation. As confirmed by TEM imaging and DLS analysis, purified *P. acnes* EVs had spherical vesicular structures with an average diameter of 42.5 ± 7.0 nm (Fig. 1). The size of *P. acnes* EV is mostly in the range of 18 to 38 nm, accounting for 91.8% of the total. Structural characteristics of *P. acnes* EVs conformed to the general description of EVs.^24^

### Effect of *P. acnes* EVs on RAW264.7 cells

**Fig. 2:**
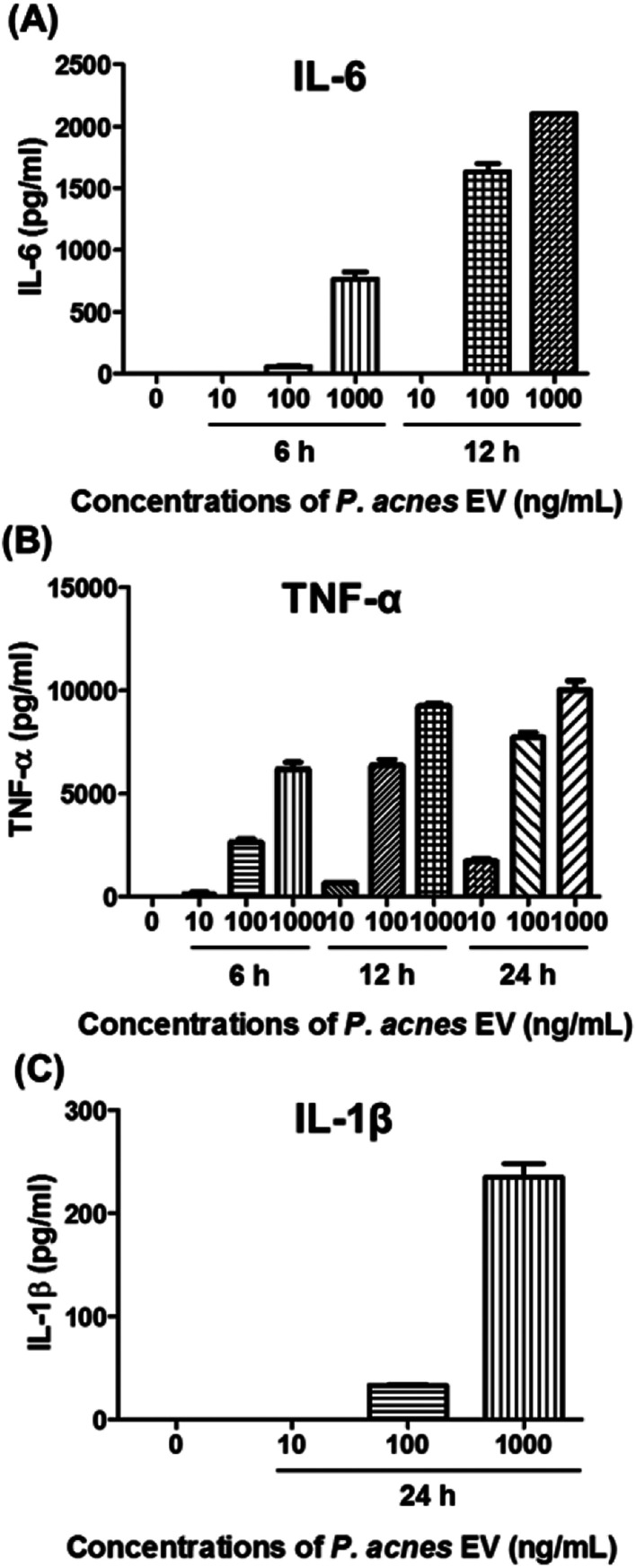
Pro-inflammatory effects of *P. acnes* EV on RAW264.7 cells. RAW264.7 cells were incubated with 10–1000 ng mL^−1^*P. acnes* EVs for 6–24 h. (A–C) The expression of IL-6, TNF-α, and IL-1β was examined by ELISA. Results were obtained in triplicate.

*P. acnes* can stimulate pro-inflammatory cytokines in host cell lines.^25–29^ so we investigated whether *P. acnes* EVs possessed bioactive molecules that could induce inflammatory responses in immune cells. When RAW264.7 cells were incubated with various concentrations of *P. acnes* EVs, increased expression of the pro-inflammatory cytokines IL-6, TNF-α, and IL-1β was induced in the presence of *P. acnes* EVs, and increased in a dose- and time-dependent manner (Fig. 2). This suggests that *P. acnes* EVs possess bioactive molecules that can act as potent immune modulators. Furthermore, the immunogenicity of *P. acnes* EVs provides an explanation for how the non-motile *P. acnes* can induce *P. acnes*-specific immune responses from distant tissues.

### Lipid identification in *P. acnes* and *P. acnes* EVs

**Fig. 3:**
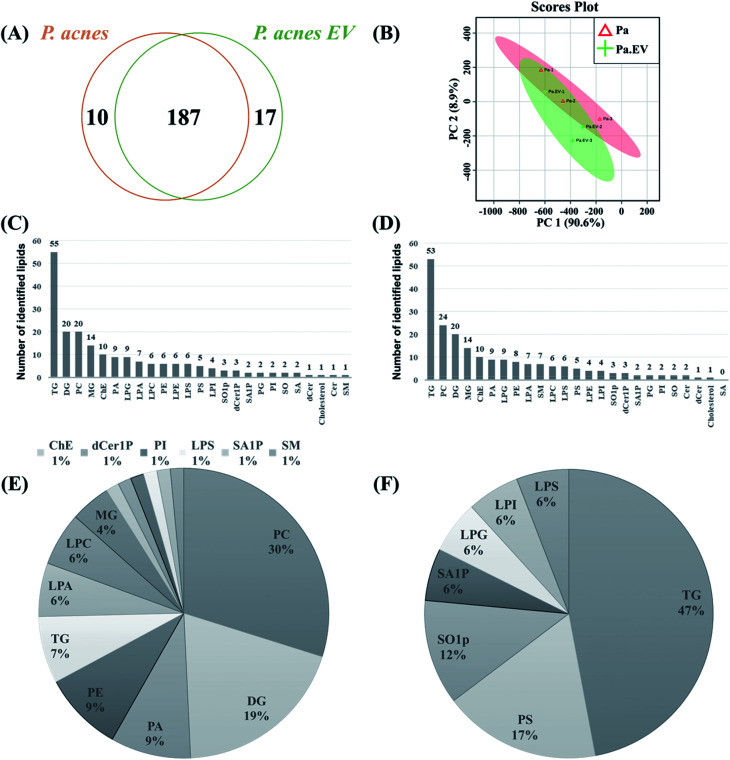
Lipid profiles of *P. acnes* and *P. acnes* EVs (A) Venn diagram and (B) principal component analysis of identified lipids in *P. acnes* and *P. acnes* EVs. Pa and Pa.EV stand for *P. acnes* and *P. acnes* EV, respectively. The numbers of identified lipids from (C) *P. acnes* and (D) *P. acnes* EVs arranged by amount. (E and F) Pie charts of differentially up- and down-regulated lipids, respectively, in *P. acnes* EVs compared to *P. acnes*.

A total of 214 lipids were identified by lipidomic analysis of *P. acnes* and *P. acnes* EVs. Among the lipids, 187 (87.38%) were identified in both *P. acnes* and *P. acnes* EVs (Fig. 3A), which contained 204 and 197 lipids, respectively As shown in the PCA analysis (Fig. 3B) and ESI Table 1,† although the lipid profiles of *P. acnes* and *P. acnes* EVs mostly overlapped, relative lipid quantities of the lipid molecules in two groups showed distinct differences (ESI Tables 1 and 2†). Among the lipid classes, TG was the most common lipid class in both groups, followed by DG, PC, MG, and ChE in *P. acnes* and PC, DG, MG, and ChE in *P. acnes* EVs (Fig. 3C and D). Among the lipids belonging to the TG class, in *P. acnes*, TG(50 : 1), TG(50 : 0), TG(50 : 2), TG(54 : 3), TG(48 : 1), and TG(52 : 2) were the most abundant in descending order. In *P. acnes* EVs, TG(54 : 3), TG(50 : 1), TG(50 : 0), TG(50 : 2), TG(52 : 2), and TG(48 : 1) were the most abundant in descending order. But throughout the lipid classes, PS(28 : 0), MG(18 : 0), MG(16 : 0), PG(28 : 0) were the most abundant lipids in both *P. acnes* and *P. acnes* EVs (ESI Table 1†). Neutral lipids were predominant in both groups.

Relative quantification of the lipids in the two groups showed 84 differentially regulated lipids (DRLs), including 67 that identified more (fold change > 1.5, *p*-value < 0.05), and 17 that identified less (fold change < 0.67, *p*-value < 0.05; ESI Table 2†) in *P. acnes* EVs compared to *P. acnes*. *P. acnes* EVs contained substantially more PCs, DGs, PAs, PEs, LPAs, LPCs, and MGs than *P. acnes*, and fewer PSs, SO1Ps, SA1Ps, LPGs, LPIs, and LPSs. Lipids in the TG class showed individually different quantities in the two groups, and the amount of total TG was markedly decreased in *P. acnes* EVs (Fig. 3E and F). Throughout all lipid classes, PC(30 : 1) showed the most dramatic difference, with a level 1210-fold higher in *P. acnes* EVs than in *P. acnes*; this was followed by PE(34 : 2), PA(30 : 1), PC(32 : 2), PC(34 : 2), PC(38 : 1), PA(34 : 1), PC(28 : 0), PA(32 : 1), PC(38 : 2), among others (ESI Tables 2†) There were also DRLs that showed little difference between *P. acnes* and *P. acnes* EVs. The lipids with a difference of less than 5% in the two groups were as follows: TG(46 : 0), LPE(20 : 0), LPS(18 : 1), ChE(20 : 1), TG(58 : 0), TG(60 : 3), TG(56 : 2), LPS(20 : 1), TG(50 : 2), LPG(20 : 3), TG(52 : 0), TG(50 : 0), TG(54 : 5), and TG(52 : 5) (ESI Table 1†).

## Discussion

*P. acnes* is a lipophilic commensal bacterium that resides on the skin and in the gastrointestinal tract.^1,2^*P. acnes* is usually nonpathogenic and has relatively low virulence, but rarely, it can exhibit serious pathogenicity.^5^ Although *P. acnes* has recently received attention as an opportunistic pathogen in various diseases, studies on its pathogenic role have mainly focused on acne.^30–32^ The pathology of acne vulgaris is a multifactorial process including increased sebum production, altered follicular keratinization, inflammation, and bacterial colonization of the pilosebaceous unit.^33,34^

Up to now, most studies on the mechanism of sebum production have focused only on host lipids in the sebaceous glands, where *P. acnes* is expected to be present.^35,36^ Although the potential role of *P. acnes*-derived lipids in host lipid metabolism and inflammation remains unclear, the most recent information regarding lipid composition, especially fatty acid, of *P. acnes* was published in 1967.^37^ We therefore performed *P. acnes* lipidomic analysis for the first time, to establish a basis for research on lipids derived from *P. acnes*.

Recent studies have revealed that both Gram-positive and Gram-negative bacteria secrete EVs.^24^ It is anticipated that even in tissues that bacteria do not directly reach, bacteria-specific host responses can be induced by EVs,^11^ which contain a large number of biomolecules from their bacterium of origin.^6,21^ To test this hypothesis, we purified *P. acnes* EVs from culture medium and incubated them with a mouse macrophage cell line. *P. acnes* EVs induced the expression of the pro-inflammatory cytokines IL-6, TNF-α, and IL-1β in the macrophages, consistent with the stimulation of pro-inflammatory cytokines by *P. acnes* in keratinocytes,^25,26^ sebocytes,^27,28^ and monocytic cells.^29^ The results suggest that *P. acnes* EVs contain bioactive molecules and may affect distant tissues on behalf of the non-motile *P. acnes*.

To identify potentially bioactive molecules in *P. acnes* EVs, we analyzed the lipidomes of *P. acnes* and *P. acnes* EVs. Interestingly, from a total of 214 identified lipids, the lipid profiles mostly overlapped but the lipid quantities showed distinct differences. In both groups, some lipids such as PS(28 : 0), MG(18 : 0), MG(16 : 0), and PG(28 : 0) were the most abundant and other lipids such as TG(46 : 0), LPE(20 : 0), LPS(18 : 1), ChE(20 : 1), TG(58 : 0), TG(60 : 3), TG(56 : 2), LPS(20 : 1), TG(50 : 2), LPG(20 : 3), TG(52 : 0), TG(50 : 0), TG(54 : 5), and TG(52 : 5) showed little difference. The reason why these lipids showed similar configurations in both groups may be because they are essential molecules to support structure, necessary for membrane construction, or they have similar functions. *P. acnes* EVs contained substantially more PCs, DGs, PAs, PEs, LPAs, LPCs, and MGs than *P. acnes* and fewer PSs, SO1Ps, SA1Ps, LPGs, LPIs, and LPSs. Distinctively, *P. acnes* EVs possessed a markedly reduced amount of TG compared to *P. acnes* and possessed dramatically elevated amounts of PC(30 : 1), PE(34 : 2), PA(30 : 1), PC(32 : 2), PC(34 : 2), PC(38 : 1), among other lipids. (ESI Table 2†). This suggests that *P. acnes* EVs may have distinctly different roles than the bacteria itself.

By identifying these constituents, we hope to ultimately elucidate the mechanism of action of *P. acnes* and its EVs in the bio-physiology and the pathology of *P. acnes*. We anticipate that a variety of factors contribute to the mechanisms of action of EVs. First, the biomolecules on the surface of EVs can factor into the mechanism of action. Surface biomolecules may bind to receptors or ligands on the surface of the target cell, or membrane-bound enzymes on EVs may play a role as well. In this process, unlike a single molecule interaction, various membrane biomolecules on the EV surface can interact with their targets simultaneously. Second, biomolecules inside the EVs may also factor into the mechanism of action. After endocytosis, biomolecules such as proteins, carbohydrates, lipids, and nucleic acids in the EVs may function or interact with counterpart molecules in target cells or act as cofactors. Unlike a single molecule interaction, as a cargo ship, EVs can simultaneously transfer numerous biomolecules at high local concentrations. For penetration, we assume that lipids or proteins may mediate the intrusion process. We hope that this data will enable study of previously unreported roles for *P. acnes*-derived lipids and *P. acnes* EVs, and provide clues towards unknown pathogenic mechanisms.

## Conclusions

In this study, we have performed a comparative lipidomic analysis of *P. acnes* and *P. acnes* EVs for the first time, and propose that *P. acnes* EVs may act as pro-inflammatory modulators in the host, stimulating IL-6, TNF-α, and IL-1β expression. A total of 214 lipids were successfully analyzed by optimized UPLC and MRM. Statistical data analysis indicated that *P. acnes* EVs had distinct lipid composition compared to *P. acnes*. We expect these findings to provide useful clues towards understanding the biological and pathophysiological mechanisms of *P. acnes* and developing clinical applications such as vaccines, diagnostics and therapeutics.

## Conflicts of interest

There are no conflicts to declare.

## Abbreviations

TG: Triacylglycerol
DG: Diacylglycerol
MG: Monoacylglycerol
ChE: Cholesterolester
PC: Phosphatidylcholine
LPC: Lysophosphatidylcholine
PE: Phosphatidylethanolamine
LPE: Lysophosphatidylethanolamine
PS: Phosphatidylserine
LPS: Lysophosphatidylserine
PG: Phosphatidylglycerol
LPG: Lysophosphatidylglycerol
PI: Phosphatidylinositol
LPI: Lysophosphatidylinositol
PA: Phosphatidic acid
LPA: Lysophosphatidic acid
So: Sphingosine
So1P: Sphingosine-1-phosphate
Sa: Sphinganine
Sa1P: Sphinganine-1-phosphate
SM: Sphingomyelin
Cer: Ceramide
dCer: Dihydroceramide
Cer1P: Ceramide-1-phosphate
dCer1P: Dihydroceramide-1-phosphate

## Supplementary Material

RA-008-C7RA13769A-s001
