# Supplementary material for: Comparative lipidomic profiling of the human commensal bacterium Propionibacterium acnes and its extracellular vesicles
Source: RSC Adv. 2018 Apr 23;8(27):15241–7. doi: 10.1039/c7ra13769a (PMC9080044; doi:10.1039/c7ra13769a)
Supplement: RA-008-C7RA13769A-s001 [file RA-008-C7RA13769A-s001.pdf]

Electronic Supplementary Material (ESI) for RSC Advances.
This journal is © The Royal Society of Chemistry 2018

Electronic Supplementary Information

Comparative Lipidomic Profiling of the Human Commensal Bacterium Propionibacterium acnes and Its Extracellular Vesicles

Jinseong Jeon[a†], Seung Cheol Park[b†], Jin Her[c], Jae Won Lee[b], Jin-Kwan Han[a], Yoon-Keun Kim[d], Kwang Pyo Kim[b] and Changill Ban[*c]

[a] *Department of Life Sciences, Pohang University of Science and Technology (POSTECH), 77, Cheongam-Ro, Nam-Gu, Pohang, Gyeongbuk, Republic of Korea, 37673.*

[b] *Department of Applied Chemistry, College of Applied Science, Kyung Hee University, Yongin, Republic of Korea, 17104.*

[c] *Department of Chemistry, Pohang University of Science and Technology (POSTECH), 77, Cheongam-Ro, Nam-Gu, Pohang, Gyeongbuk, Republic of Korea, 37673.*

[d] *Myeoungdo Medical, Woori Technology Building, World Cup buk-ro 56-gil 9, Mapo-gu, Seoul, Republic of Korea, 03923.*

Supplementary table 1. Identified lipids in *P. acnes* EV and *P. acnes*.

	P. acnes EV			*P. acnes*		
	P. acnes EV-1	*P. acnes* EV-2	*P. acnes* EV-3	*P. acnes* -1	*P. acnes* -2	*P. acnes* -3
TG(42:0)	0.3172	0.2647	0.2855	0.5130	0.4559	0.5059
TG(44:0)	1.6806	1.3109	1.6027	2.6902	2.3432	2.2357
TG(44:1)	0.4398	0.4079	0.4599	0.8924	0.8482	0.8640
TG(46:0)	4.0872	3.8035	4.0869	4.4333	4.0126	4.1492
TG(46:1)	1.1643	1.1073	1.1868	2.5543	2.3802	2.5672
TG(46:2)	0.2787	0.3002	0.2995	0.5500	0.5675	0.4898
TG(48:0)	1.3127	1.3441	1.2993	1.5674	1.4512	1.4830
TG(48:1)	9.0582	9.0506	9.9802	12.1707	11.4147	11.7140
TG(48:2)	1.6051	1.6494	1.9005	3.0092	2.7914	2.8091
TG(48:3)	0.4393	0.4476	0.4584	1.4022	1.1726	1.2366
TG(50:0)	12.7770	12.1687	12.8853	13.2924	11.6570	11.9954
TG(50:1)	13.8153	12.5544	15.2997	20.6210	19.1421	19.8126
TG(50:2)	11.4086	12.5538	12.7587	12.2915	11.5546	12.3059
TG(50:3)	2.9061	2.9232	3.2848	2.8832	2.8741	2.9140
TG(50:4)	0.1271	0.1552	0.1143	0.2651	0.2618	0.2743
TG(52:0)	0.0042	0.0038	0.0072	ND	ND	ND
TG(52:0)	5.2903	4.7309	5.0122	5.1168	4.6051	5.0091
TG(52:1)	3.8539	3.0947	4.0214	4.7799	4.4423	4.7605
TG(52:2)	10.1855	11.0357	13.0046	9.5696	9.0624	9.4000
TG(52:3)	6.6449	7.5463	8.0244	4.7918	4.8680	5.0522
TG(52:4)	1.2920	1.3380	1.4123	1.4596	1.3814	1.4526
TG(52:5)	0.1893	0.1332	0.1817	0.1533	0.1411	0.1871
TG(54:0)	5.6526	4.1744	5.3569	7.2505	6.2980	6.5935
TG(54:1)	2.1972	1.6794	2.3850	2.7185	2.3858	2.5473
TG(54:2)	4.5418	3.7706	4.8832	4.3005	3.9046	4.0699
TG(54:3)	23.1530	21.7111	25.2237	12.0614	11.4741	12.0306
TG(54:4)	4.7470	4.3592	4.8539	3.1663	3.0536	3.0441
TG(54:5)	2.4820	2.2279	2.5052	2.3522	2.1859	2.4077
TG(54:6)	1.5644	1.2866	1.5667	1.7833	1.7220	1.6946
TG(54:7)	0.3973	0.4704	0.3460	0.3038	0.3300	0.2270
TG(58:0)	0.1584	0.1782	0.1516	0.1781	0.1801	0.1416
TG(58:1)	0.3809	0.3727	0.3313	0.4820	0.4187	0.2664
TG(58:2)	0.4500	0.4861	0.3935	0.4654	0.4804	0.2352
TG(58:3)	0.1286	0.1630	0.1272	0.1411	0.1239	0.0791
TG(58:4)	0.1092	0.1702	0.1400	0.1041	0.0871	0.0493
TG(58:5)	0.1146	0.1240	0.1006	0.0957	0.0891	0.0365
TG(58:6)	0.0103	0.0160	0.0094	0.0117	0.0117	0.0165
TG(58:8)	0.0132	0.0312	0.0096	ND	ND	ND

TG(60:0)	0.0540	0.0585	0.0463	0.0671	0.0477	0.0304
TG(60:1)	0.1127	0.1166	0.0815	0.1002	0.1106	0.0576
TG(60:2)	0.0862	0.0902	0.0810	0.0867	0.0944	0.0494
TG(60:3)	0.0583	0.0627	0.0541	0.0821	0.0555	0.0388
TG(60:4)	0.0182	0.0237	0.0222	ND	ND	ND
TG(62:0)	0.0084	0.0088	0.0071	0.0142	0.0169	0.0061
TG(62:3)	0.0103	0.0160	0.0163	0.0103	0.0052	0.0034
TG(64:0)	0.0070	0.0084	0.0084	0.0102	0.0124	0.0081
TG(56:0)	0.8133	0.8671	0.7452	1.1734	1.0411	0.6806
TG(56:1)	0.9760	1.0947	0.8761	1.4788	1.3294	0.7555
TG(56:2)	1.1187	1.2211	0.9040	1.3178	1.2305	0.7155
TG(56:3)	0.8551	0.9409	0.8513	0.5565	0.5371	0.3543
TG(56:4)	0.2343	0.3091	0.2746	0.1388	0.1498	0.0692
TG(56:5)	0.2729	0.3483	0.3225	0.1972	0.1515	0.0821
TG(56:6)	0.0912	0.0980	0.0820	0.0486	0.0389	0.0401
TG(52:1)	ND	ND	ND	0.0056	0.0064	0.0074
TG(54:6)	ND	ND	ND	0.0576	0.0457	0.0519
TG(60:2)	ND	ND	ND	0.0149	0.0092	0.0081
TG(62:0)	ND	ND	ND	0.0094	0.0089	0.0117
TG(64:1)	ND	ND	ND	0.0081	0.0076	0.0042
SA1P(d14:1)	0.6047	0.6314	0.8550	1.3534	1.1377	1.3641
SA1P(d18:1)	1.4882	3.5429	4.1734	0.1224	0.0408	0.2172
SO1P(d14:1)	0.1965	0.1924	0.1871	0.5210	0.4699	0.8010
SO1P(d16:1)	0.4939	0.5100	0.5175	0.3417	0.3880	0.4668
SO1P(d18:1)	2.0492	2.4138	2.7224	3.6134	3.5792	4.9541
dCer1P(d18:1-14:0)	0.9282	1.0542	0.7043	0.5547	0.7020	1.2191
dCer1P(d18:1-18:3)	0.2320	0.4036	0.4739	0.6336	0.6446	0.4871
dCer1P(d18:1-20:3)	1.4530	0.8675	1.1609	0.6819	0.4592	0.5461
dCer(d14:1-16:1)	8.3425	7.3795	6.7826	12.4427	5.4084	8.9888
Cholesterol	0.0980	0.1320	0.2060	0.1470	0.1230	0.1110
ChE(16:1)	0.1000	0.1690	0.1270	0.1990	0.1460	0.1650
ChE(16:0)	0.0660	0.0740	0.0620	0.0540	0.0480	0.0410
ChE(18:2)	0.0680	0.1020	0.1100	0.0460	0.0370	0.0280
ChE(18:1)	0.0850	0.1860	0.1830	0.1000	0.0670	0.0940
ChE(18:0)	0.0360	0.0420	0.0720	0.0190	0.0230	0.0140
ChE(20:2)	0.0100	0.0070	0.0090	0.0090	0.0090	0.0110
ChE(20:1)	0.0120	0.0100	0.0200	0.0240	0.0120	0.0070
ChE(20:0)	0.0200	0.0230	0.0310	0.0220	0.0170	0.0120
ChE(24:1)	0.0120	0.0200	0.0250	0.0370	0.0220	0.0280
ChE(24:0)	0.0180	0.0320	0.0240	0.0360	0.0240	0.0420
DG(30:0)	16.5740	14.6230	16.6000	1.9660	1.9490	2.0650
DG(30:1)	0.0600	0.0540	0.0530	0.0140	0.0120	0.0120

DG(32:0)	15.2780	12.7360	15.5000	6.6640	5.5900	6.7970
DG(32:1)	0.0970	0.1270	0.1110	0.0230	0.0210	0.0310
DG(32:2)	0.1170	0.1100	0.1130	0.0070	0.0060	0.0060
DG(34:0)	9.7220	8.0660	9.8100	7.8530	6.5640	8.0000
DG(34:1)	0.1440	0.2240	0.1920	0.0620	0.0550	0.0660
DG(34:2)	0.0940	0.1270	0.0930	0.0190	0.0220	0.0230
DG(34:3)	0.0300	0.0400	0.0320	0.0040	0.0050	0.0060
DG(36:0)	5.6940	4.1390	5.3600	4.6720	4.0430	4.7970
DG(36:1)	0.0560	0.0520	0.0620	0.0170	0.0170	0.0200
DG(36:2)	0.0740	0.1070	0.0850	0.0320	0.0300	0.0270
DG(36:3)	0.0390	0.0550	0.0430	0.0160	0.0140	0.0150
DG(36:4)	0.0260	0.0330	0.0260	0.0130	0.0100	0.0120
DG(38:0)	0.1190	0.1050	0.1070	0.0930	0.0850	0.1160
DG(38:4)	0.0380	0.0350	0.0390	0.0330	0.0340	0.0350
DG(40:0)	0.0310	0.0250	0.0350	0.0230	0.0290	0.0270
DG(40:4)	0.1020	0.0910	0.1010	0.0900	0.0760	0.0940
DG(42:0)	0.0330	0.0330	0.0350	0.0230	0.0200	0.0230
DG(42:4)	0.0570	0.0500	0.0540	0.0540	0.0460	0.0530
MG(14:0)	2.1370	1.7690	3.2120	1.6900	1.6040	1.2940
MG(16:0)	211.6750	189.3520	266.6670	147.3870	195.4170	101.7060
MG(18:4)	0.5480	0.4860	0.8420	0.6240	0.4710	0.5490
MG(18:3)	0.9490	1.0830	1.2790	0.7320	0.9290	0.7300
MG(18:2)	1.1170	1.1900	1.5580	0.7700	0.9290	0.6380
MG(18:1)	8.4260	7.1760	10.9090	7.3870	7.7920	7.2010
MG(18:0)	261.4210	238.8890	318.7880	210.8010	210.0000	155.2900
MG(20:2)	1.6290	1.5600	2.0300	0.8150	0.9960	0.9900
MG(20:1)	10.9140	10.7410	16.2420	10.2090	11.7500	10.3410
MG(20:0)	2.9440	2.5230	2.6790	2.0520	1.8210	2.0380
MG(22:5)	7.6650	7.1300	9.5150	4.4250	4.9170	4.4370
MG(22:2)	0.6700	0.5790	0.8180	0.5710	0.7290	0.6350
MG(22:1)	0.5010	0.5000	0.6610	0.5160	0.5540	0.4230
MG(24:1)	0.3410	0.2500	0.4460	0.2600	0.2830	0.2480
PA(28:0)	0.7171	0.3124	0.3682	0.0264	0.0210	0.0100
PA(28:5)	0.1105	0.1584	0.1113	0.0777	0.0650	0.1288
PA(30:0)	15.4264	7.9562	8.1026	2.8220	2.4667	0.7834
PA(30:1)	2.2984	1.5474	1.4205	0.0289	0.0217	0.0128
PA(32:0)	0.4767	0.2343	0.2133	0.0113	0.0107	0.0112
PA(32:1)	0.5310	0.2073	0.2795	0.0096	0.0078	0.0118
PA(34:1)	0.6977	0.2526	0.3154	0.0066	0.0039	0.0124
PA(36:1)	0.9651	0.3759	0.2656	0.0061	0.0039	ND
PA(38:1)	0.7326	0.1777	0.1533	0.0042	ND	ND
LPA(14:0)	13.9781	11.5753	15.9036	5.4682	6.4734	6.6536
LPA(16:0)	7.9197	6.7808	7.0683	4.4816	4.0902	3.8356

LPA(16:1)	2.8540	2.5411	1.7028	1.2358	1.1884	1.2133
LPA(18:0)	2.1350	1.5103	1.5944	1.4833	1.4332	0.7730
LPA(18:1)	1.4307	1.1781	1.3092	0.7759	0.7536	0.8943
LPA(18:2)	0.4489	0.4452	0.5703	0.6254	0.5668	0.5812
LPA(20:2)	1.2226	0.9315	0.8273	1.0903	1.0676	1.0098
LPC(16:0)	0.0243	0.0204	0.0204	0.0196	0.0163	0.0180
LPC(16:1)	0.0034	0.0032	0.0036	0.0007	0.0006	0.0006
LPC(18:0)	0.0106	0.0115	0.0106	0.0081	0.0067	0.0073
LPC(18:1)	0.0124	0.0111	0.0120	0.0020	0.0023	0.0017
LPC(18:2)	0.0031	0.0026	0.0033	0.0014	0.0011	0.0017
LPC(24:1)	0.0016	0.0020	0.0016	0.0014	0.0010	0.0008
PC(28:0)	0.0119	0.0119	0.0128	0.0003	0.0003	0.0002
PC(28:1)	0.0138	0.0121	0.0132	0.0010	0.0009	0.0007
PC(30:0)	0.0305	0.0303	0.0358	0.0021	0.0018	0.0020
PC(30:1)	0.3844	0.3918	0.4345	0.0004	0.0004	0.0002
PC(30:2)	0.0051	0.0043	0.0048	ND	ND	ND
PC(32:0)	0.0140	0.0152	0.0153	0.0020	0.0028	0.0025
PC(32:1)	0.0389	0.0400	0.0448	0.0028	0.0027	0.0029
PC(32:2)	0.0318	0.0327	0.0330	0.0005	0.0005	0.0005
PC(34:0)	0.0071	0.0073	0.0086	0.0017	0.0020	0.0016
PC(34:1)	0.0912	0.0918	0.0972	0.0042	0.0043	0.0054
PC(34:2)	0.1551	0.1469	0.1745	0.0023	0.0026	0.0026
PC(34:3)	0.0028	0.0029	0.0029	0.0004	0.0003	0.0004
PC(36:0)	0.0098	0.0094	0.0112	0.0021	0.0019	0.0025
PC(36:1)	0.0206	0.0239	0.0278	0.0009	0.0012	0.0010
PC(36:2)	0.0471	0.0497	0.0559	0.0023	0.0020	0.0023
PC(36:3)	0.0113	0.0114	0.0129	0.0007	0.0008	0.0009
PC(36:4)	0.0076	0.0074	0.0084	0.0008	0.0007	0.0008
PC(36:5)	0.0017	0.0019	0.0017	ND	ND	ND
PC(38:1)	0.1660	0.1605	0.1648	0.0028	0.0025	0.0026
PC(38:2)	0.0803	0.0755	0.0807	0.0024	0.0020	0.0024
PC(38:3)	0.0077	0.0078	0.0080	0.0005	0.0005	0.0005
PC(38:4)	0.0011	0.0011	0.0014	0.0002	0.0003	0.0003
PC(40:1)	0.0049	0.0051	0.0055	ND	ND	ND
PC(40:2)	0.0032	0.0036	0.0037	ND	ND	ND
PE(34:0)	0.0002	0.0003	0.0002	0.0001	0.0001	0.0001
PE(34:1)	0.0019	0.0017	0.0019	0.0002	ND	0.0001
PE(34:2)	0.0170	0.0161	0.0159	0.0001	0.0001	0.0001
PE(36:1)	0.0003	0.0005	0.0005	0.0001	0.0002	0.0001
PE(36:2)	0.0017	0.0020	0.0015	0.0001	ND	0.0001
PE(36:3)	0.0006	0.0007	0.0003	0.0001	0.0001	0.0001
PE(36:4)	0.0006	0.0007	0.0007	ND	ND	ND
PE(36:5)	0.0003	0.0003	0.0002	ND	ND	ND

LPE(18:2)	0.0006	0.0010	0.0007	0.0010	0.0011	0.0012
LPE(20:0)	0.0036	0.0032	0.0053	0.0045	0.0048	0.0034
LPE(20:1)	0.0008	0.0012	0.0007	0.0009	0.0012	0.0015
LPE(22:0)	0.0058	0.0053	0.0070	0.0075	0.0064	0.0053
LPE(18:1)	ND	ND	ND	0.0008	0.0020	0.0011
LPE(20:2)	ND	ND	ND	0.0019	0.0012	0.0014
PG(28:0)	172.8810	24.8230	14.9180	125.2940	44.8430	0.8820
PG(28:4)	0.3170	0.2420	0.3010	0.5230	0.3550	0.2750
LPG(16:0)	0.5720	0.5970	0.5760	0.5650	0.9740	0.8210
LPG(18:0)	0.5350	0.5510	0.5590	0.2720	0.5120	0.2950
LPG(18:1)	0.4950	0.6190	0.6330	0.3340	0.7410	0.3460
LPG(18:2)	2.5520	5.5510	3.8290	1.4900	4.3310	3.0360
LPG(20:2)	0.1510	0.2220	0.2430	0.2720	0.3490	0.4870
LPG(20:3)	0.5350	0.4210	0.4690	0.4460	0.4770	0.4770
LPG(22:0)	0.2360	0.2860	0.3260	0.2310	0.5060	0.3340
LPG(22:2)	0.1990	0.2310	0.1880	0.3340	0.3430	0.3660
LPG(22:3)	0.6620	0.4280	0.3800	0.4020	0.4450	0.2300
PI(32:0)	0.3000	0.4341	0.2958	ND	0.0496	ND
PI(30:0)	1.4634	1.5015	1.3921	0.0575	0.0515	0.0558
LPI(14:0)	0.3525	0.6801	0.6377	0.5769	0.5498	0.4242
LPI(16:0)	0.5550	0.6539	0.6833	0.6474	0.4084	0.4659
LPI(18:0)	0.1915	0.4553	0.3974	0.4712	0.3473	0.4280
LPI(18:3)	0.1740	0.3135	0.2044	0.5160	0.4855	0.5909
PS(28:0)	468.5908	139.2523	151.8193	590.9091	356.8075	68.1818
PS(28:1)	0.0942	ND	ND	0.1159	0.0330	ND
PS(28:4)	0.1455	0.0808	0.0831	0.3545	0.2504	0.3056
PS(32:5)	0.0718	0.0157	0.0258	0.1025	0.0776	0.1217
PS(34:3)	0.2869	0.2065	0.1907	0.5636	0.3584	0.6136
LPS(14:0)	0.6220	0.6571	0.5944	0.8966	0.7494	1.1568
LPS(16:0)	0.3780	0.4199	0.3992	0.3289	0.2530	0.1962
LPS(16:1)	3.2622	4.4231	3.3976	2.9178	2.5059	1.7839
LPS(18:1)	1.4055	1.0064	1.2450	1.3899	1.3310	1.0508
LPS(18:2)	0.3293	0.2808	0.2799	0.4642	0.4799	0.5890
LPS(20:1)	0.7652	0.4583	0.4257	0.6844	0.6856	0.2818
SO(d16:1)	1.4430	1.2550	0.7870	1.8760	1.3580	2.6220
SO(d18:1)	2.7610	1.6880	1.1350	1.8410	2.1330	3.6300
Cer(d18:1-16:0)	0.2180	0.2590	0.4070	ND	ND	ND
Cer(d18:1-22:0)	0.0710	0.0650	0.1080	ND	ND	ND
SM(d18:1-14:0)	0.0090	0.0090	0.0090	ND	ND	ND
SM(d18:1-16:0)	0.1960	0.2140	0.2070	0.0040	0.0120	0.0090
SM(d18:1-18:0)	0.0380	0.0430	0.0380	ND	ND	ND
SM(d18:1-18:1)	0.0020	0.0030	0.0040	ND	ND	ND
SM(d18:1-20:0)	0.0400	0.0490	0.0430	ND	ND	ND

SM(d18:1-22:0)	0.0690	0.0690	0.0680	ND	ND	ND
SM(d18:1-24:0)	0.0340	0.0550	0.0390	ND	ND	ND
Cer(d18:1-18:2)	ND	ND	ND	0.0440	0.0320	0.0230
SA(d16:0)	ND	ND	ND	0.7540	0.7980	0.6530
SA(d18:0)	ND	ND	ND	0.3150	0.2650	0.2880

Table S1. The numbers were the calculated values of peak area for each lipid molecule detected by mass spectroscopy divided by internal standard.

Supplementary table 2. Differentially regulated lipids (p-value < 0.05)

Up-regulated in *P. acnes* EV (fold change > 1.5) compared to *P. acnes*

No.	Compound Name	Fold changes	Log_2(Fold changes)	P-value	$-log_{10}$(P-value)
1	ChE(18:2)	2.523	1.335	1.54.E-02	1.813
2	dCer1P(d18:1-20:3)	2.063	1.045	2.98.E-02	1.525
3	DG(30:0)	7.993	2.999	2.90.E-05	4.538
4	DG(30:1)	4.395	2.136	4.70.E-05	4.328
5	DG(32:0)	2.284	1.192	1.08.E-03	2.968
6	DG(32:1)	4.467	2.159	7.05.E-04	3.152
7	DG(32:2)	17.895	4.162	8.14.E-07	6.089
8	DG(34:1)	3.060	1.614	5.87.E-03	2.232
9	DG(34:2)	4.906	2.295	1.76.E-03	2.754
10	DG(34:3)	6.800	2.766	7.35.E-04	3.134
11	DG(36:1)	3.148	1.655	2.30.E-04	3.639
12	DG(36:2)	2.989	1.580	3.85.E-03	2.415
13	DG(36:3)	3.044	1.606	3.18.E-03	2.497
14	DG(36:4)	2.429	1.280	2.61.E-03	2.584
15	DG(42:0)	1.530	0.614	6.30.E-04	3.200
16	LPA(14:0)	2.229	1.157	4.29.E-03	2.368
17	LPA(16:0)	1.755	0.811	1.33.E-03	2.878
18	LPA(16:1)	1.951	0.964	2.85.E-02	1.546
19	LPA(18:1)	1.617	0.693	4.23.E-03	2.373
20	LPC(16:1)	5.368	2.425	2.11.E-05	4.676
21	LPC(18:1)	5.917	2.565	2.00.E-05	4.698
22	LPC(18:2)	2.143	1.100	4.11.E-03	2.386
23	LPC(24:1)	1.625	0.700	3.94.E-02	1.405
24	LPS(16:0)	1.539	0.622	2.57.E-02	1.590
25	MG(18:2)	1.654	0.726	3.36.E-02	1.473
26	MG(20:2)	1.863	0.898	6.99.E-03	2.156
27	MG(22:5)	1.764	0.819	9.03.E-03	2.044
28	PA(28:0)	24.350	4.606	2.43.E-02	1.614
29	PA(30:0)	5.185	2.374	2.91.E-02	1.536
30	PA(30:1)	83.065	6.376	3.19.E-03	2.497
31	PA(32:0)	27.840	4.799	2.46.E-02	1.610
32	PA(32:1)	34.856	5.123	2.83.E-02	1.548
33	PA(34:1)	55.271	5.788	4.08.E-02	1.389
34	PC(28:0)	45.750	5.516	2.45.E-06	5.612
35	PC(28:1)	15.038	3.911	1.77.E-05	4.753
36	PC(30:0)	16.373	4.033	7.41.E-05	4.130

37	PC(30:1)	1210.700	10.242	1.34.E-05	4.874
38	PC(32:0)	6.096	2.608	1.32.E-05	4.881
39	PC(32:1)	14.726	3.880	2.92.E-05	4.534
40	PC(32:2)	65.000	6.022	9.66.E-08	7.015
41	PC(34:0)	4.340	2.118	2.63.E-04	3.580
42	PC(34:1)	20.158	4.333	1.38.E-06	5.860
43	PC(34:2)	63.533	5.989	4.42.E-05	4.354
44	PC(34:3)	7.818	2.967	7.57.E-07	6.121
45	PC(36:0)	4.677	2.226	1.56.E-04	3.808
46	PC(36:1)	23.323	4.544	3.78.E-04	3.423
47	PC(36:2)	23.136	4.532	4.87.E-05	4.312
48	PC(36:3)	14.833	3.891	2.90.E-05	4.538
49	PC(36:4)	10.174	3.347	2.16.E-05	4.666
50	PC(38:1)	62.190	5.959	6.95.E-08	7.158
51	PC(38:2)	34.779	5.120	1.37.E-06	5.862
52	PC(38:3)	15.667	3.970	1.25.E-07	6.902
53	PC(38:4)	4.500	2.170	8.98.E-04	3.047
54	PE(34:0)	2.333	1.222	1.61.E-02	1.792
55	PE(34:1)	15.714	3.974	2.78.E-05	4.556
56	PE(34:2)	163.330	7.352	1.13.E-06	5.948
57	PE(36:1)	3.250	1.700	1.58.E-02	1.801
58	PE(36:2)	20.800	4.379	3.52.E-04	3.454
59	PE(36:3)	5.333	2.415	2.26.E-02	1.645
60	PI(30:0)	26.438	4.725	1.67.E-06	5.778
61	SA1P(d18:1)	24.197	4.597	2.23.E-02	1.651
62	SM(d18:1-16:0)	24.680	4.625	4.26.E-06	5.371
63	TG(52:3)	1.510	0.595	3.71.E-03	2.431
64	TG(54:3)	1.969	0.978	3.75.E-04	3.426
65	TG(56:3)	1.828	0.871	4.83.E-03	2.316
66	TG(56:4)	2.077	1.055	1.25.E-02	1.905
67	TG(56:5)	1.942	0.957	2.51.E-02	1.600

Down-regulated in *P.acnes* EV (fold change < 0.67) compared to *P. acnes*

No.	Compound Name	Fold changes	Log_2(Fold changes)	P-value	$-log_{10}$(P-value)
1	TG(44:0)	0.632	-0.662	7.35.E-03	2.134
2	TG(48:2)	0.599	-0.740	5.68.E-04	3.245

3	LPG(22:2)	0.593	-0.755	9.06.E-04	3.043
4	SO1P(d18:1)	0.592	-0.757	2.84.E-02	1.547
5	TG(42:0)	0.588	-0.766	1.01.E-03	2.996
6	LPS(18:2)	0.581	-0.785	7.26.E-03	2.139
7	TG(46:2)	0.546	-0.872	5.87.E-04	3.231
8	SA1P(d14:1)	0.542	-0.883	5.59.E-03	2.253
9	TG(44:1)	0.502	-0.994	2.66.E-05	4.576
10	TG(50:4)	0.495	-1.014	4.37.E-04	3.360
11	TG(46:1)	0.461	-1.117	3.15.E-05	4.501
12	PS(34:3)	0.445	-1.167	2.74.E-02	1.563
13	LPI(18:3)	0.435	-1.203	4.69.E-03	2.329
14	PS(32:5)	0.375	-1.413	4.30.E-02	1.367
15	TG(48:3)	0.353	-1.502	2.78.E-04	3.555
16	PS(28:4)	0.340	-1.557	5.52.E-03	2.258
17	SO1P(d14:1)	0.321	-1.638	1.70.E-02	1.770
